# Cascadable Current-Mode First-Order All-Pass Filter Based on Minimal Components

**DOI:** 10.1155/2013/859784

**Published:** 2013-06-13

**Authors:** Jitendra Mohan, Sudhanshu Maheshwari

**Affiliations:** ^1^Department of Electronics & Communication Engineering, Jaypee Institute of Information Technology, Noida 201304, India; ^2^Department of Electronics Engineering, Z.H. College of Engineering and Technology, Aligarh Muslim University, Aligarh 202002, India

## Abstract

A novel current-mode first-order all-pass filter with low input and high output impedance feature is presented. The circuit realization employs a single dual-X-second-generation current conveyor, one grounded capacitor, and one grounded resistor, which is a minimum component realization. The theoretical results are verified using PSPICE simulation program with TSMC 0.35 **μ**m CMOS process parameters.

## 1. Introduction

Current-mode circuit design using current conveyor has received a considerable attention owning to its potential advantages such as wider dynamic range, greater linearity, wide bandwidth, simple circuitry, and low power consumption [1]. Considering these advantages of current conveyor, recently several current mode first-order all-pass filters employing different types of current conveyor such as second-generation current conveyor [2–4], four terminal floating nullor [5], third-generation current conveyor [6], differential voltage current conveyor [7, 8], current differencing buffered amplifier [9], current operational amplifier [10], and dual-X second-generation current conveyor [11, 12] have been reported. These reported filters reveal some useful features depending on the individual topology as summarized in Table 1. The comparison between the proposed circuit and the previously reported circuits is based on the use of number of active elements, number of grounded passive components, and low input and high output impedance feature(s). In general, the input impedance should be lower in comparison to the output impedance to avoid loading problem while cascading such current-mode circuits to form larger system.

In this paper, a novel cascadable current-mode (CM) first-order all-pass filter is proposed. The circuit uses a dual-X second generation multioutput current conveyor (DX-MOCCII), a grounded resistor, and a grounded capacitor, which is ideal for IC implementation. The circuit offers low-input impedance and high-output impedance feature and also free from matching constraints. Nonideal gain and parasitic effects of the DX-MOCCII on the transfer function of the proposed filter are also analysed.

## 2. The Proposed Circuit

**Figure 1:**
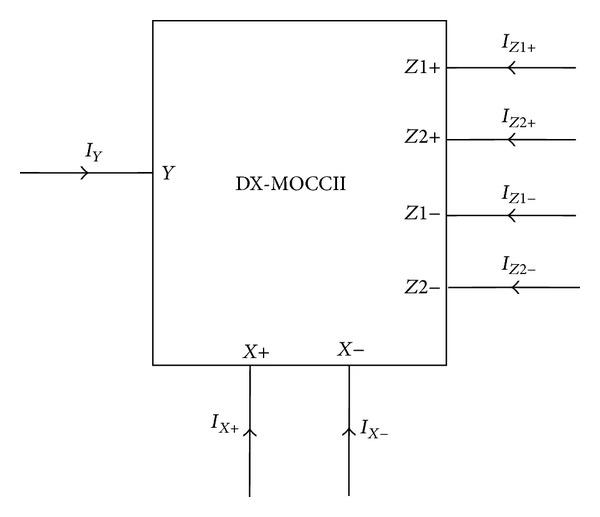
Symbol of DX-MOCCII.

Dual-X second-generation current conveyor [13] is a useful and versatile active element, which has found several applications in analog signal processing [14–18]. The DX-MOCCII symbol is shown in Figure 1 and is characterized by the following port relationships:

(1) $$\begin{array}{c} \left[\begin{array}{c} I_{Y}\\ V_{X+}\\ V_{X-}\\ I_{Z1+}\\ I_{Z2+}\\ I_{Z1-}\\ I_{Z2-} \end{array}\right]=\left[\begin{array}{ccc} 0 & 0 & 0\\ 1 & 0 & 0\\ -1 & 0 & 0\\ 0 & 1 & 0\\ 0 & -1 & 0\\ 0 & 0 & 1\\ 0 & 0 & 1 \end{array}\right]\left[\begin{array}{c} V_{Y}\\ I_{X+}\\ I_{X-} \end{array}\right], \end{array}$$

where the suffixes refer to the respective terminals. The active element is characterized by high input impedance at the *Y* terminal, high output impedance at the *Z*1+, *Z*2+, *Z*1−, and *Z*2− terminals, and low impedance at the *X*+ and *X*− terminals.

**Figure 2:**
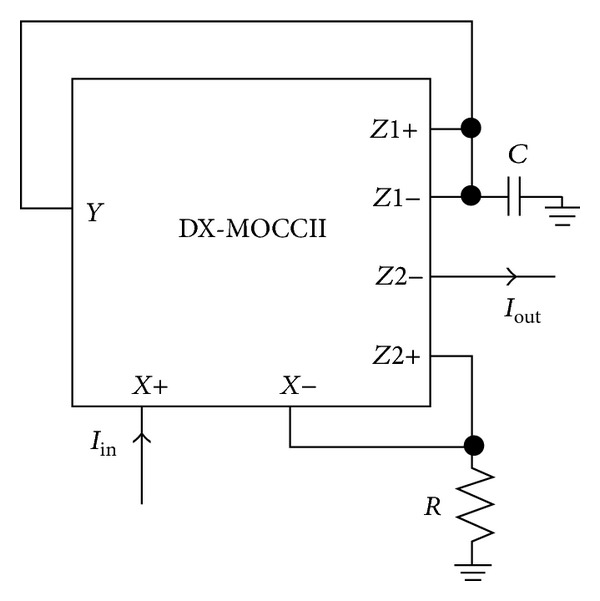
Proposed cascadable current-mode first-order all-pass filter.

The proposed current-mode (CM) first-order all-pass filter employing a DX-MOCCII, a grounded capacitor, and a grounded resistor is shown in Figure 2. Routine analysis of the circuit, using (1), yields the following transfer function:

(2) $$\begin{array}{c} \frac{I_{\text{out}}}{I_{\text{in}}}=-\left(\frac{s-\left(1/CR\right)}{s+\left(1/CR\right)}\right). \end{array}$$

The frequency-dependent phase response of (2) is

(3) $$\begin{array}{c} \Phi =-2\;\tan^{-1}\left(\omega RC\right). \end{array}$$

From (3), it can be seen that the proposed circuit can provide a phase shift between 0° and −180° at output terminal (*I*_out_).

The salient features of the proposed circuit are the use of single active element, two grounded passive components, and providing low input and high output impedance. As all the passive components used are in grounded form, it is suitable for integrated circuit implementation and also reduces the associated parasitic effects [19].

By interchanging the resistor (*R*) with a capacitor (*C*) in Figure 2, an additional circuit can be derived from the proposed circuit. However, the use of capacitor at the *X*− terminal degrades the high frequency operation [20].

## 3. Nonideal Analysis and Parasitic Effects

### 3.1. Non-Ideal Analysis

Taking the nonidealities of the DX-MOCCII into account, the port relationship of the voltage and current terminals of the active element can be rewritten as

(4) $$\begin{array}{c} \left[\begin{array}{c} I_{Y}\\ V_{X+}\\ V_{X-}\\ I_{Z1+}\\ I_{Z2+}\\ I_{Z1-}\\ I_{Z2-} \end{array}\right]=\left[\begin{array}{ccc} 0 & 0 & 0\\ \beta_{1} & 0 & 0\\ -\beta_{2} & 0 & 0\\ 0 & \alpha_{1} & 0\\ 0 & -\alpha_{2} & 0\\ 0 & 0 & \alpha_{3}\\ 0 & 0 & \alpha_{4} \end{array}\right]\left[\begin{array}{c} V_{Y}\\ I_{X+}\\ I_{X-} \end{array}\right]. \end{array}$$

Here, *α*_1_ and *α*_2_ are the current transfer gains from *X*+ terminal to *Z*1+ and *Z*2+ terminals, *α*_3_ and *α*_4_ are the current transfer gains from *X*− terminal to *Z*1− and *Z*2− terminals, respectively, and *β*_1_ and *β*_2_ are the voltage transfer gains from input to *X*+ and *X*− terminals, respectively. More specifically, *α*_1_ = (1 − *ε*_1_),  *α*_2_ = (1 − *ε*_2_),  *α*_3_ = (1 − *ε*_3_),  *α*_4_ = (1 − *ε*_4_),  *β*_1_ = (1 − *δ*_1_), and *β*_2_ = (1 − *δ*_2_), where *ε* is the current transfer error (tracking error) and *δ* is the voltage transfer error (tracking error) of the DX-MOCCII. However, these transfer gains differ from unity by the voltage and current tracking errors of the DX-MOCCII. 

The proposed circuit is reanalyzed by taking the tracking errors of the nonideal MO-DXCCII into account, and the modified current transfer function is given as

(5) $$\begin{array}{c} \frac{I_{\text{out}}}{I_{\text{in}}}=-\alpha_{2}\alpha_{4}\left(\frac{s-\left(\beta_{2}\alpha_{1}/CR\alpha_{2}\right)}{s+\left(\beta_{2}\alpha_{3}/CR\right)}\right). \end{array}$$

Equation (5) reveals that the nonidealities do affect the filter gain and the pole frequency as well as the zero frequency. Assuming matched current transfer gains (*α*_1_ and *α*_3_) the phase characteristics would not be affected. The sensitivities of pole frequency (*ω*_o_) and gain (*H*) with respect to active and passive components are derived from (5). These are as follows:

(6) $$\begin{array}{c} S_{C,R}^{\omega_{o}}=-1,\;\;S_{\alpha_{3},\beta_{2}}^{\omega_{o}}=1,\;\;S_{\alpha_{1},\alpha_{2},\alpha_{4},\beta_{1}}^{\omega_{o}}=0,\\ S_{C,R}^{H}=0,\;\;S_{\alpha_{1},\alpha_{3},\beta_{1},\beta_{2}}^{H}=0,\;\;S_{\alpha_{2},\alpha_{4}}^{H}=1. \end{array}$$

From the results, it is evident that the sensitivities are within unity in magnitude, thus ensuring a low sensitivity performance.

### 3.2. Parasitic Effects

Next study is carried on the effect of device parasitics on the performance of the proposed circuit. The various parasitics are a low value parasitic serial resistance *R*_*X*_ at *X* the terminal *Y* exhibits a high value parasitic resistance *R*_*Y*_ in parallel with low value capacitor *C*_*Y*_, and the terminals *Z* exhibit a high value parasitic resistance *R*_*Z*_ in parallel with low value capacitance *C*_*Z*_. The main among these are the *Y* and *Z* terminals parasitic capacitances and the *X* terminal's parasitic resistances. A reanalysis of the proposed circuit yields the modified transfer function as

(7) $$\begin{array}{c} \frac{I_{\text{out}}}{I_{\text{in}}}=-\left(\frac{sRC^{\prime }-sRC_{Z2+}-1}{s^{2}RR_{X}C^{\prime }C_{Z2+}+s\left(RC^{\prime }+R_{X}C^{\prime }+RC_{Z2+}\right)+1}\right), \end{array}$$

where *C*′ = *C* + *C*_*Z*1−_ + *C*_*Z*1+_.

From (7), the effect of capacitance *C*_*Z*2+_ becomes nonnegligible at very high frequencies. Most of the parasitic capacitances get absorbed with the external grounded capacitor, as are in shunt with it. Also the parasitic resistance gets absorbed with the external grounded resistor, as it is in series with it. Such a merger will cause a slight deviation in circuit parameters, which can be corrected by predistorting the passive element values used in the circuit. 

## 4. Simulation Results

**Figure 3:**
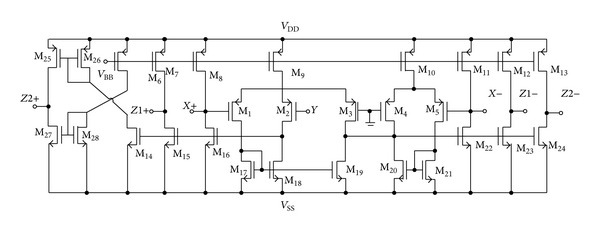
CMOS implementation of DX-MOCCII.

**Figure 4:**
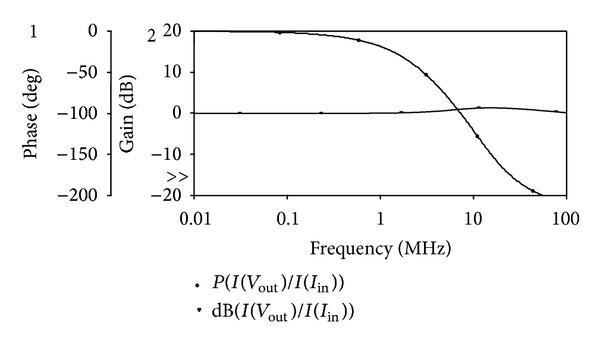
Simulated gain and phase responses of the all-pass filter.

**Figure 5:**
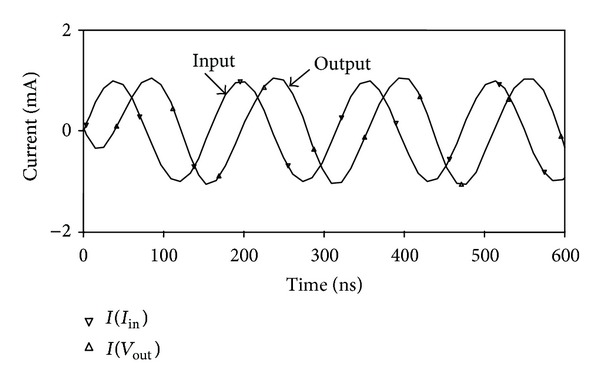
Time-domain input and output responses of all-pass filter.

**Figure 6:**
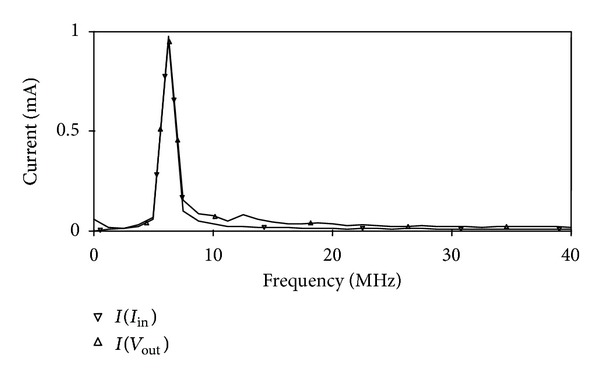
Fourier spectrum of the input and output.

**Figure 7:**
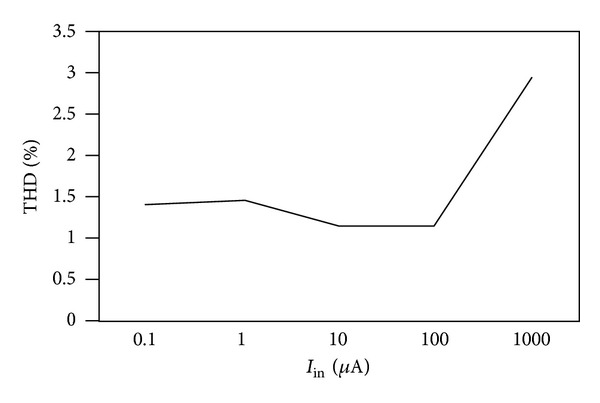
THD variation at output (*I*_out_) with sinusoidal signal amplitude at 6.36 MHz.

To demonstrate the performance of the proposed circuit, the PSPICE simulation program is used. In the simulation, the TSMC 0.35 **µ**m CMOS process parameters were used. The CMOS implementation of DX-MOCCII is shown in Figure 3 [13]. The aspect ratios of the CMOS transistors of the DX-MOCCII are listed in Table 2. DC supply voltages of ±1.8 V and biasing voltage of *V*_*BB*_ = −0.7 V were used. The proposed circuit of Figure 2 was designed with *R* = 1 kΩ and *C* = 25 pF to obtain a pole frequency of 6.36 MHz. The gain and phase responses are shown in Figure 4, where a phase shift of 90° at a pole frequency of 6.27 MHz is obtained, which is close to the theoretical designed value. The time-domain input and output responses of the circuit at the pole frequency are shown in Figure 5. Also, the Fourier spectrum of input signal and output signal is shown in Figure 6. Next, the amplitude of the input sinusoidal signal is varied from 0.1 **µ**A to 1000 **µ**A, and the total harmonic distortion (THD) curve is plotted at a pole frequency of 6.36 MHz and is shown in Figure 7. 

## 5. Conclusion

In this paper, a new current-mode cascadable all-pass filter is presented. The proposed circuit uses single DX-MOCCII, a grounded resistor, and a grounded capacitor, which is the minimum component realization for an active RC filter circuit. The circuit requires no matching constraints and low active and passive sensitivities and employs grounded passive components only, which makes it suitable for integrated circuit implementation. The circuit also exhibits the feature of low-input impedance and high-output impedance. The PSPICE simulation results of the proposed circuit are in good agreement with the theoretical results. 

## Figures and Tables

**Table 1:** Comparison of various current-mode all-pass filters.

References	No. of active elements	Single active element	No. of resistors and capacitors	All-grounded passive elements	Low input impedance	High output impedance	Component matching constraint
Higashimura and Fukui [2]	1-CCII	Yes	4	No	No	Yes	Yes
Higashimura [5]	1-FTFN	Yes	3	No	No	No	Yes
Toker et al. [9]	1-CDBA	Yes	2	No	No	Yes	No
Maheshwari and Khan [6]	1-CCIII	Yes	2	No	No	No	No
Kilinç and Çam [10]	1-COA	Yes	2	No	No	Yes	No
Minaei and Ibrahim [7]	1-DVCC	Yes	3	No	No	Yes	Yes
Khan et al. [3]	2-MOCCII	No	2	Yes	No	Yes	No
Maheshwari [8]	1-DVCC	Yes	2	Yes	No	Yes	No
Minaei and Yuce [4]	2-DOCCII	No	2	Yes	Yes	Yes	No
Minaei and Yuce [11]	1-DXCCII	Yes	4	Yes	No	Yes	Yes
Beg et al. [12]	1-DX-MOCCII	Yes	4	Yes	No	Yes	Yes
Proposed Circuit	1-DX-MOCCII	Yes	2	Yes	Yes	Yes	No

**Table 2:** Aspect ratios of the transistors.

Transistors	*W* (**µ**m)	*L* (**µ**m)
M_1_-M_2_	1.4	0.7
M_3_–M_5_	2.8	0.7
M_17_-M_18_	2.4	0.7
M_19_–M_21_	4.8	0.7
M_6_–M_16_, M_22_–M_28_	9.6	0.7
